# Determinants of Chromatin Organization in Aging and Cancer—Emerging Opportunities for Epigenetic Therapies and AI Technology

**DOI:** 10.3390/genes15060710

**Published:** 2024-05-29

**Authors:** Rogerio M. Castilho, Leonard S. Castilho, Bruna H. Palomares, Cristiane H. Squarize

**Affiliations:** 1 Laboratory of Epithelial Biology, Department of Periodontics and Oral Medicine, School of Dentistry, University of Michigan, Ann Arbor, MI 48109-1078, USA; lcastilh7@gmail.com (L.S.C.); csquariz@umich.edu (C.H.S.); 2 Rogel Cancer Center, University of Michigan, Ann Arbor, MI 48109-1078, USA; 3 Oral Diagnosis Department, Piracicaba School of Dentistry, State University of Campinas, Piracicaba 13414-903, Sao Paulo, Brazil; brunahddpalomares@gmail.com

**Keywords:** histone modifications, cancer epigenetics, histone code, aging, artificial intelligence

## Abstract

This review article critically examines the pivotal role of chromatin organization in gene regulation, cellular differentiation, disease progression and aging. It explores the dynamic between the euchromatin and heterochromatin, coded by a complex array of histone modifications that orchestrate essential cellular processes. We discuss the pathological impacts of chromatin state misregulation, particularly in cancer and accelerated aging conditions such as progeroid syndromes, and highlight the innovative role of epigenetic therapies and artificial intelligence (AI) in comprehending and harnessing the histone code toward personalized medicine. In the context of aging, this review explores the use of AI and advanced machine learning (ML) algorithms to parse vast biological datasets, leading to the development of predictive models for epigenetic modifications and providing a framework for understanding complex regulatory mechanisms, such as those governing cell identity genes. It supports innovative platforms like CEFCIG for high-accuracy predictions and tools like GridGO for tailored ChIP-Seq analysis, which are vital for deciphering the epigenetic landscape. The review also casts a vision on the prospects of AI and ML in oncology, particularly in the personalization of cancer therapy, including early diagnostics and treatment optimization for diseases like head and neck and colorectal cancers by harnessing computational methods, AI advancements and integrated clinical data for a transformative impact on healthcare outcomes.

## 1. Introduction

**Figure 1:**
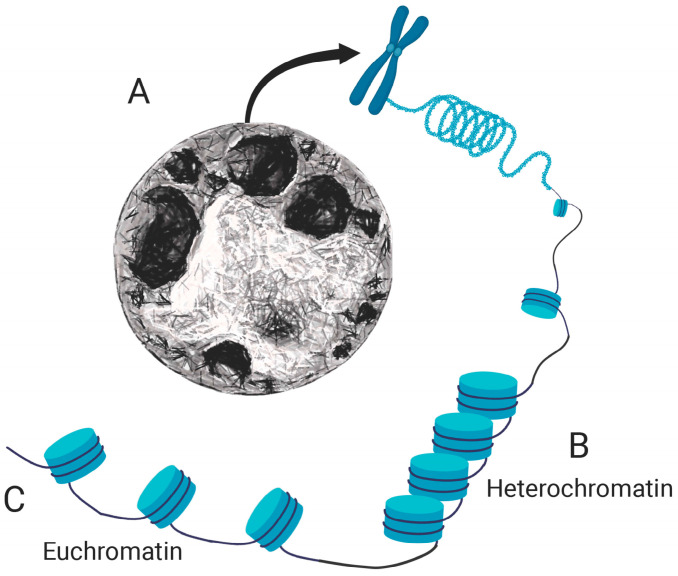
Schematic illustration of cellular chromatin organization. (**A**) Overview of chromatin organization showing the distribution of different chromatin states. (**B**) Detail of heterochromatin, indicating regions of tightly packed DNA commonly associated with gene silencing and structural support. (**C**) Detail of euchromatin, illustrating areas where DNA is less condensed, typically corresponding to transcriptionally active regions. The figure was generated using a combination of Midjourney image generator (nuclear images) and BioRender (diagram).

The chromatin comprises a highly organized complex of DNA and proteins, predominantly histones, that condenses to form chromosomes during cell division. Various mechanisms, including histone modifications, DNA methylation and the action of chromatin remodeling complexes, modulate the level of chromatin compaction. It is evident that the organization of the chromatin is not static, instead, it is highly dynamic, shifting among the chromatin states, euchromatin and heterochromatin (Figure 1A–C). The compaction state of the chromatin is a fundamental process that regulates cellular function, behavior and identity, where the degree of compaction modulates the accessibility of genes to the transcriptional system.

Histones systematically package extensive strands of DNA into a more compact and manageable form, making it possible for the genetic material to fit within the confines of the nucleus [1,2]. Moreover, histone modifications, such as acetylation and methylation, are crucial for maintaining cellular homeostasis, tissue regeneration and healing by regulating gene expression and chromatin structure (Table 1) [3]. 

The complex regulatory interplay language that directs the functional outcomes of chromatin structure and gene expression dynamics is termed the histone code [39]. This code encompasses an array of post-translational modifications, including acetylation, methylation, phosphorylation and ubiquitination, which can signal chromatin compaction or relaxation, influencing the accessibility of DNA to the transcriptional machinery.

Deciphering the histone code opens up new avenues in the fields of epigenetics and personalized medicine, which can lead to transformative discoveries. This code not only maintains the cell identity and the cell function within a functional tissue and organism but also has the potential to pass down non-DNA sequence-based information across generations, which environmental factors can influence. This phenomenon is known as transgenerational epigenetic inheritance [40]. It can lead to heritable changes that may impact an offspring’s health and disease risk. The dysregulation of this code has been tied to numerous illnesses, including cancer, neurological disorders and cardiovascular diseases. A deeper comprehension of the histone code opens the door to precision medicine, where treatments can be tailored to specific epigenetic alterations in individual patients [41,42].

In this review, we will discuss the latest understanding of the structure of chromatin and its implications in aging and cancer, along with the emerging opportunities in artificial intelligence technology to facilitate a better understanding of the histone code.

## 2. Chromatin Organization and Its Structures

### 2.1. Heterochromatin

Heterochromatin is a densely packed type of chromatin (Figure 1B) with fewer mobile nucleosome clusters than euchromatin [43,44]. The compact nature of heterochromatin suggests a state of genetic inactivity, which was later demonstrated in cases where chromosomal rearrangements led to gene silencing when euchromatic genes were relocated near heterochromatic regions. There are two main types of heterochromatin: constitutive and facultative. Constitutive heterochromatin is consistent across cell types. It is composed of repetitive elements at centromeres and telomeres, whereas the facultative type forms in areas with developmentally regulated genes and varies among different cell types [45,46,47].

Constitutive heterochromatin features marks such as H3K9 trimethylation (H3K9me3). This modification, conveyed by specific histone methyltransferases, aids the maintenance of the condensed chromatin conformation and affects accurate chromosome segregation and structural integrity. The formation and spread of this heterochromatin type are reinforced by feedback mechanisms involving RNAi components and proteins, like the Heterochromatin Protein 1 (HP1), which binds to H3K9me3, promoting compaction [48,49]. Notably, HP1 is found in three different isoforms, α, β and γ, whereas HP1α and HP1β are commonly found in heterochromatin; HP1γ is found in heterochromatin and euchromatin [50,51]. The presence of HP1γ and H3K9 methylation has also been associated with transcription elongation and telomere stability [52,53].

Facultative heterochromatin regulation involves Polycomb group (PcG) proteins that leave epigenetic marks, such as the trimethylation of H3K27 (H3K27me3). Additionally, facultative heterochromatin is established at specific genomic domains, such as imprinted loci and *HOX* gene clusters, where it modulates gene expression during development. Non-coding RNAs, originating from Polycomb response elements, interact with PcG proteins and RNAi components to maintain heterochromatin states [54,55].

### 2.2. Euchromatin

Euchromatin functions as a more accessible segment of chromatin, embodying a less dense structure that is essential for active transcription (Figure 1C). This open state is characterized by a looser association of the nucleosomes due to histone acetylation, which weaken the interaction of histone tails and enable easy access of transcription machinery to the DNA [56]. Regulatory systems within euchromatin ensure that only the particular genes are active at specific times and locations, maintaining the delicate balance of gene expression required for stem cell pluripotency and cell fate, function and behavior [57,58,59,60,61,62].

Special transcription factors, known as pioneer factors, are instrumental regulatory functions, especially because they recognize and bind to DNA sequences within heterochromatin and trigger chromatin remodeling and transitioning to euchromatin. FOXA and OCT4 are two pioneer factors that play important roles in gene regulation. FOXA helps modify the chromatin structure during development by displacing histone H1, which allows the enhancer nucleosome to be more easily accessed. It participates in the recruitment of RNA polymerase II (Pol II) and the histone variant H2A.Z [63]. Its expression affects endoderm-derived tissues, such as the pancreas, liver, thyroid, kidney, prostate and lung, and mutations are present in epithelial-origin cancers [64,65]. OCT4, on the other hand, is involved in regulating pluripotency in embryonic stem cells and is one of the four transcription factors used to induce pluripotent cells [66,67]. Following the pioneer factors, settler factors come into play. Settler factors are transcription factors that bind to the chromatin after it has been opened up by pioneer factors, further stabilizing the open chromatin state and promoting gene transcription [68]. An example of a settler factor is the Nuclear Respiratory Factor 1 (NRF1), which settles into promoter regions of genes involved in mitochondrial function after pioneer factors such as FOXA1 have altered the chromatin landscape [69].

Promoters, located at the beginning of genes, are crucial for initiating transcription and often feature well-positioned nucleosomes and regions devoid of nucleosomes, known as nucleosome-depleted regions. They facilitate the binding of transcription factors and the transcriptional machinery. Enhancers are found at variable distances from promoters. They enhance gene expression by binding to specific transcription factors, contributing to the spatial organization of the chromatin by looping mechanisms. They are often associated with activating histone modifications to facilitate transcription regulation [58,70,71,72].

Histone modifications play a significant role in the function of euchromatin, with certain modifications like H3K4me3 marking promoter regions near transcription start sites, H3K27ac indicating active enhancers and gene bodies, and H3K36me decorating the gene bodies of actively transcribed genes [73]. These post-translational modifications of histones serve as signals that help recruit chromatin-modifying complexes and transcription factors, thus influencing gene activity.

The regulatory systems of euchromatin are further regulated by histone variants, which can replace standard histones within the nucleosome and confer distinct properties to chromatin. Variants like H2A.Z and H3.3 are incorporated into nucleosomes throughout the cell cycle and are associated with active transcription [74,75]. Furthermore, non-coding RNAs (ncRNAs) interact with chromatin and can influence chromatin looping, as well as the overall organization and function of the euchromatin [76,77].

## 3. Chromatin Imbalance in Aging and Cancer

### 3.1. Chromatin State in Stem Cell

Stem cells are unique in their ability to self-renew and differentiate into various specialized cell types. These properties underpin their critical role in development, regeneration, cancer development and aging. Stem cell fate regulation is complex and controlled by a combination of extracellular signals, transcriptional programs and chromatin organization [78,79]. In stem cells, chromatin is generally maintained in a more open and relaxed state, which facilitates the expression of genes necessary for maintaining pluripotency and cellular quiescence, while compaction of the chromatin is associated with differentiation [80,81].

Histone modifications affect the chromatin states, playing a significant role in stem cell identity and differentiation. This includes the methylation, acetylation, phosphorylation and ubiquitylation of histones responsible for the physical properties of chromatin [82]. For instance, trimethylation of H3K4 (H3K4me3) and acetylation of H3K27 (H3K27ac) are marks typically associated with active gene transcription and are enriched at genes that maintain stem cell programs [83,84]. Conversely, the trimethylation of H3K27 (H3K27me3), catalyzed by the Polycomb Repressive Complex 2 (PRC2), is commonly involved with repression and silencing of genes associated with differentiation in stem cells [85,86] (Table 2). Additionally, DNA methylation has a profound impact on stem cell maintenance, while CpG island methylation within promoter regions typically leads to gene silencing [87,88].

**Figure 2:**
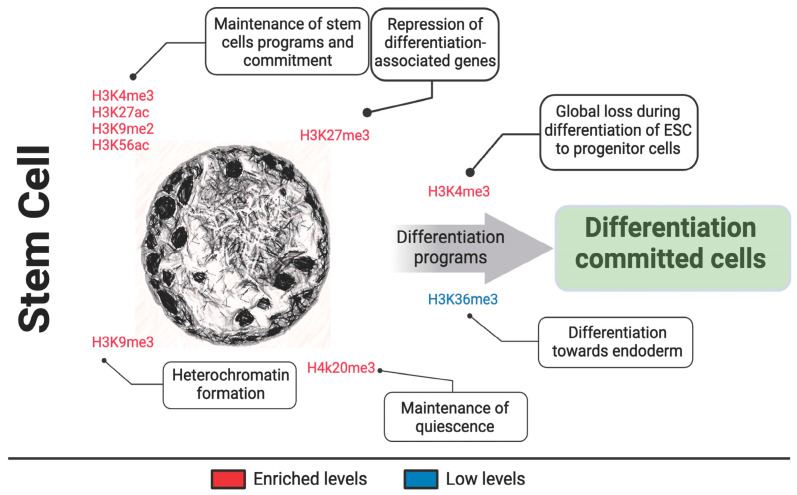
Histone modification landscape in stem cell chromatin. This schematic illustrates the chromatin environment of a stem cell, highlighting key histone modifications such as acetylation and methylation involved in heterochromatin formation and the maintenance of stem cell-associated programs. The diagram provides a visual summary of the sites and types of modifications reported in stem cell chromatin. The figure was generated using a combination of Midjourney image generator (nuclear images) and BioRender (diagram).

The maintenance and self-renewal of stem cells also require functional chromatin remodelers. Chromatin remodelers are specialized protein complexes that reposition nucleosomes, influencing chromatin accessibility. ATP-dependent remodeling complexes, such as switch/sucrose non-fermentable (SWI/SNF), play crucial roles in the maintenance of ESCs by facilitating chromatin accessibility at loci that promote pluripotency and inhibiting the expression of differentiation-inducing genes. The balanced action of these complexes is essential for the fine-tuning of gene expression required for stem cell self-renewal and the maintenance of pluripotency [99]. Indeed, if the SWI/SNF fails to function correctly due to mutations, it will lead to additional intracellular genomic instability, affecting transcription and replication of cells, ultimately leading to premature aging and even cancer [100,101,102] (Figure 2).

### 3.2. Chromatin Imbalances in Cancer

Chromatin imbalance results from disruptions in the standard switch between heterochromatin and euchromatin [103]. These disruptions can affect gene expression and genomic stability, leading to various pathologies. During disease development and progression, such imbalances can impair the proper function of genes by either promoting gene silencing or causing unscheduled gene activation. The dysregulation of genetic pathways can contribute to a range of diseases, from cancer, where chromatin modifications may lead to the activation of oncogenes and silencing of tumor suppressor genes, to neurodegenerative diseases like Huntington’s, Alzheimer’s and ataxias, where altered chromatin affects neuronal gene expression [104,105,106]. Moreover, epigenetic changes linked to chromatin imbalance participate in the progression of autoimmune diseases and developmental disorders by altering cell differentiation and immune responses.

Similarly, chromatin imbalance may contribute to cancer development. For instance, cancer cells often exhibit global changes in chromatin organization, such as histone modifications or DNA methylation patterns, which result in a more relaxed chromatin structure, predisposing the cells to genetic instability and aberrant gene activation. Histone modifications can also contribute to cancer progression by changing the epigenetic landscape and enabling aberrant gene expression, which drives tumorigenesis (Table 3). This is the case of the tri-methylation of Histone 3 on lysine 27 (H3K27me3) mediated by enhancer of zeste homolog 2 (EZH2) [107,108,109], which acts as an epigenetic silencer of tumor suppressor genes, including *CDKN2A* and *RARβ*, among others [110,111,112]. 

Moreover, mutations in genes coding for chromatin remodeling proteins, histones, or enzymes that modify histones and DNA are common in various types of cancers like lymphomas that contain activating mutations in *EZH2* and leukemias presenting MLL-fusion proteins with the methylation of H3K79 driven by the histone methyltransferase DOTL1 [135,136]. These mutations can disrupt the delicate balance of chromatin dynamics, leading to inappropriate gene expression and the silencing of tumor suppressor genes or activation of oncogenes, ultimately contributing to oncogenesis and the proliferation of cancer cells [111]. Aberrant expression or epigenetic modulation of chromatin remodelers confers to cancer cells the ability to reprogram their genome to maintain oncogenic phenotypes. Several types of chromatin modifications have been reported in various cancers, including the acetylation of histone tails, which is generally associated with transcriptional activation and is mediated by histone acetyltransferases (HATs) [144]. Conversely, histone deacetylases (HDACs) remove acetyl groups, often leading to transcriptional repression [145]. The dysregulation of HATs and HDACs is implicated in several cancers, including hepatocellular carcinomas, colorectal and breast cancers, leukemia, lymphomas and neuroblastomas, to cite a few.

Methylation of lysine residues on histones is implicated in the chromatin imbalance, altering the active and repressive chromatin states. These modifications are regulated by enzymes such as histone methyltransferases (HMTs) and histone demethylases (HDMs). Abnormal patterns of methylation, particularly on H3K27, have been implicated in the pathogenesis of non-Hodgkin lymphomas, specifically diffuse large B-cell lymphoma and follicular lymphoma [125,146,147]. Methylation of H3K4 is driven by the fusion protein advent from the translocation of the mixed lineage leukemia (MLL) gene and is associated with the maintenance of leukemia stem cells [113,148]. Metastatic prostate cancer is found to overexpress EZH2, which plays a role in the methylation of histones and other non-histone targets [149]. Other faulty epigenetic mechanisms are also reported in cancers, including the transcriptional silencing of genes mediated by PRCs, which are found in different types of cancers like lymphomas and solid tumors, and the phosphorylation of histones, which is associated with genomic stability and cancer development [150,151].

Emerging evidence also points towards the presence of mutations in histones and their involvement in tumor formation and progression. Termed oncohistones, specific histone mutations have been implicated in driving the development and progression of several forms of cancers. These mutations often occur heterozygously and act dominantly, altering the epigenetic landscape and affecting gene expression. In exploring oncohistones, scientists have identified canonical mutations in the histone H3 tail, primarily at positions H3K27, H3G34 and H3K36. For instance, H3K27M mutations play a significant role in pediatric midline gliomas, such as diffuse intrinsic pontine gliomas (DIPG) and thalamic gliomas. Such mutations act by inhibiting the PRC2, decreasing global H3K27me3, and leading to altered gene expression profiles characteristic of these cancers [128,129,130,131,132].

**Figure 3:**
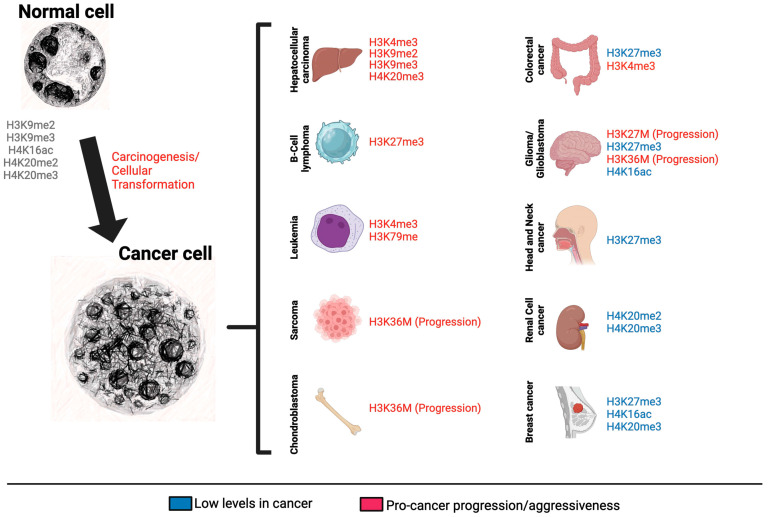
Overview of histone modifications in cancer. The schematic depicts various histone modifications that have been identified in different types of cancers. This illustration includes modifications such as methylation, acetylation and the presence of mutated histones. The figure was generated using a combination of Midjourney image generator (nuclear images) and BioRender (diagram).

Specific types of mutations found in oncohistones display a remarkable tissue specificity, contributing to the development of certain cancers. The presence of these mutations in histone genes, which are otherwise highly conserved, underscores their potent effects in oncogenesis. The H3K36M mutation is prevalent in chondroblastomas and has been associated with changes in differentiation and deregulation of cell growth, pointing to its oncogenic potential. Additionally, H3G34 mutations, which are commonly found in giant cell tumors of the bone and high-grade pediatric gliomas of the forebrain, suggest a tissue-specific influence on the histone code that promotes cancer development [131,132,133,134] (Figure 3).

### 3.3. Epigenetic Modifications in Aging

As the extent of knowledge around epigenetic alterations deepens, fresh insights emerge into the pathobiology of diseases and the mechanisms that govern the aging of organisms. Aging can result from a combination of genomic and epigenomic factors, where the integrity of the epigenetic landscape is gradually eroded due to non-mutagenic DNA repair responses to double-strand breaks (DSBs) [66]. Such erosion of the epigenetic landscape results in the acceleration of hallmarks of aging that include changes in DNA methylation patterns and histone modifications, such as lower amounts of H3K27ac and H3K56ac and higher amounts of H4K16ac and H3K122ac, along with dysregulation of *Cdkn1a*, *Myl4*, *Nlrc5*, *Mrpl55* genes, all associated with aging. Yet, the link between DSBs and the advancement of aging is not well known, having a potential explanation related to the relocalization of chromatin modifiers like ten-eleven translocation enzymes (TETs) and DNA methyltransferases (DNMTs) [152].

From a therapeutic standpoint, the reversal of the aging process, driven by the DNA repair response and increased disruption of the epigenetic landscape, may be possible through epigenetic reprogramming. Therefore, it could be achieved by leveraging the cyclic expression of the Yamanaka factors *OCT4*, *SOX2* and *KLF4* (OSK) [66,67]. This approach successfully extended the lifespan of mice displaying aging signs and reversed the aging of damaged neurons, resulting in the cure of blindness through DNA demethylation [153,154]. These findings revealed that the aged epigenetic landscape can be reverted to a youthful state. The expression of OSK factors also resulted in the reversal of age-associated mRNA expression, effectively rewinding the epigenetic clock in aging cells by up to 57%. Additionally, epigenetic markers for aging like H3K9me3 and H3K36me2, present in the kidney and muscle, respectively, were also reset to control levels. This comprehensive epigenetic reset suggests the potential for reprogramming approaches to mitigate age-related phenotypes and cellular damage.

Emerging findings on the premature manifestation of aging also provide evidence of the link between histone modifications and aging. Aging cells and accelerated aging syndromes display alterations in epigenetic marks, such as histone acetylation and methylation. Alterations in the patterns of histone modifications at H4K20me3 and H3K9me3, markers of heterochromatin and gene repression, are associated with aging [155]. Indeed, the expression of H3K9me3 is negatively correlated with memory in old mice and is enriched in aged somatic tissues, similar to the H3K4me3 levels that accumulate with age in hematopoietic stem cells [156,157,158]. On the contrary, H4K20me3 is lost during cellular aging [159,160]. During the aging process, there is a significant diminishment in the presence of the H3K9me3 modification, which is conventionally implicated in promoting the condensed packaging of DNA within the histone complex for heterochromatin formation [161,162]. Along with specific alterations of histone modifications in aging, the core histones H2A, H2B, H3 and H4 are reduced during aging [160,163,164]. The most common aging-specific histone modifications are demonstrated in Table 4.

#### Progeroid Syndromes

Chromatin imbalance and defects in nuclear lamin anchorage can result in a series of profound cellular malfunctions, often leading to rare genetic disorders collectively known as laminopathies. The nuclear lamina, composed of lamin proteins, provides structural support to the cell nucleus and also plays a critical role in the organization of chromatin within the nuclear space [178,179]. It functions as a scaffold for anchoring chromatin, thereby influencing gene expression through spatial positioning and affecting the epigenetic landscape of the cell. Disruptions in the attachment of chromatin to the nuclear lamina, often caused by mutations in the lamin A/C gene (*LMNA*), can lead to chromatin imbalance, which in turn disrupts gene regulation. This has been observed in diverse conditions such as Hutchinson-Gilford progeria syndrome, Emery-Dreifuss muscular dystrophy and certain cardiomyopathies [180].

Progeroid syndromes represent a group of rare genetic disorders with a pronounced and premature manifestation of aging-associated phenotypes and a complex connection with alterations of chromatin structure and function. Unlike the gradual onset characteristic of physiological aging, progeroid syndromes display an accelerated aging trajectory, often presenting in childhood or early adulthood. Prominent features include dermal atrophy, joint contractures and systemic manifestations such as cardiovascular complications. Among these conditions, Hutchinson-Gilford progeria syndrome (HGPS) stands as a paradigmatic example, primarily attributed to mutations in the *LMNA* gene [181,182]. Another mutation in *LMNA* genes leads to Mandibuloacral dysplasia (MAD) type A, a recessive disorder caused by an amino acid substitution (R527H) in the lamin A C-terminal immunoglobulin domain. Mutations in other genes, like *ZMPSTE24* that encodes a metallopeptidase involved in lamin A processing, are also involved in premature aging syndromes, including Mandibuloacral dysplasia type B (MADB) and Restrictive Dermopathy (RD) [180,183,184,185]. In addition, a range of progeroid conditions linked to the nuclear envelope show us just how diverse premature aging disorders can be. For example, Atypical Werner Syndrome (AWS) comes from changes in the *WRN* gene that are responsible for making a type of DNA helicase, allowing us to see how critical DNA repair is for aging. Similarly, Cockayne syndrome, tied to the genes *ERCC8* and *ERCC6*, along with Bloom syndrome, which involves a mutation in the *BLM* helicase gene, Rothmund-Thomson syndrome from *RECQL4* gene mutations and Atypical Progeria Syndrome (APS) connected to *LMNA* and *ZMPSTE24* mutations, all fall under the umbrella of progeroid laminopathies. These conditions underscore the complex and multifaceted nature of how our bodies can age prematurely at the genetic level [184]. Some of the identified histone modifications in progeroid syndromes are shown in Table 5.

Beyond the role that disruptions in chromatin balance play in sparking diseases and cancers, changes to the nuclear lamina and the subsequent organization of chromatin have unexpected and significant effects on the aging process. These alterations help us understand at a cellular level just how deeply our genes are intertwined with how we age.

HGPS exemplifies the interplay between nuclear lamina alterations and chromatin dynamics, particularly concerning histone modifications. In patients with HGPS, a mutation in the *LMNA* gene leads to the production of progerin, a deleterious version of the lamin A protein that integrates into the nuclear lamina and induces numerous nuclear abnormalities [197].

Observations in primary HGPS fibroblasts reveal that the accumulation of progerin, the mutant form of lamin A protein expressed in HGPS patients, is associated with genome-wide changes in the histone mark H3K27me3, specifically a global reduction that preempts many characteristic nuclear defects in the disease, such as aberrant nuclear shape [191]. This histone mark, typically associated with transcriptionally repressive heterochromatin, is found to be altered in patterns concurrent with gene density and gene expression changes in HGPS cells. Additionally, progerin perturbation of nuclear architecture affects the DNA–lamin A/C associations, leading to disturbances in the peripheral heterochromatin banding to the nuclear lamina [198]. These alterations in H3K27me3 are believed to precede a global loss of spatial chromatin compartmentalization, as seen in later passages of HGPS fibroblasts, which underscores the causal relationship between histone modification misregulation and chromatin structure disorganization. The dysregulation of the histone mark H3K27me3, possibly due to EZH2 downregulation, may partially explain the disrupted heterochromatin-lamina associations, leading to transcriptional misregulation and progression toward global chromatin disorganization that characterizes HGPS [199].

**Figure 4:**
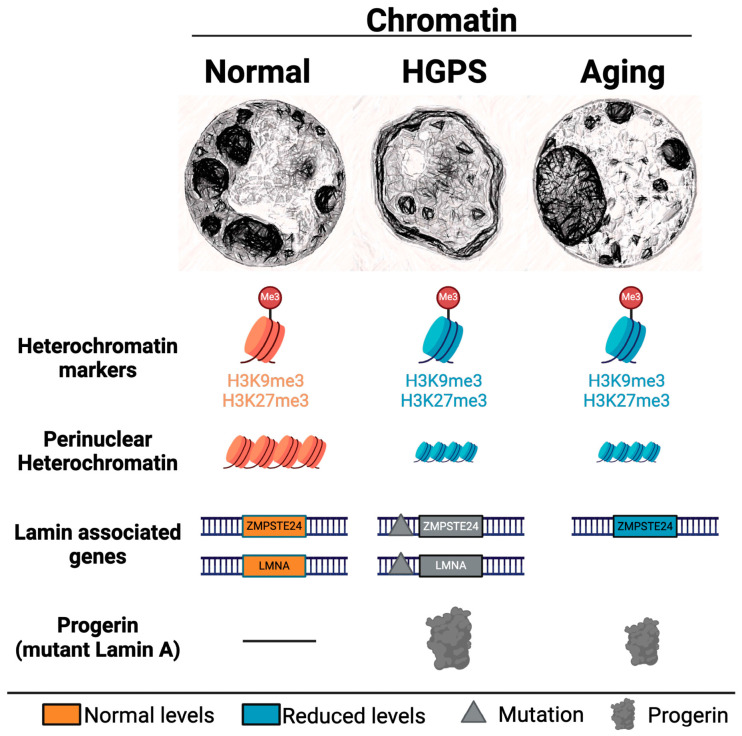
Chromatin modifications associated with aging. This illustration captures the alterations in chromatin structure and histone methylation markers, specifically H3K9me3 and H3K27me3, in relation to aging. It contrasts the expression patterns of these markers at lamin-associated genes in normal cells, cells from individuals with Hutchinson-Gilford progeria syndrome (HGPS), and cells undergoing natural aging processes. Additionally, the figure highlights the presence of wildtype and mutant ZMPSTE24 and LMNA genes and the aberrant protein progerin in HGPS and its potential accumulation in normally aging cells, offering a visual comparison of the epigenetic landscape across different cellular states associated with age-related changes. The figure was generated using a combination of Midjourney image generator (nuclear images) and BioRender (diagram).

It is interesting to note that the mechanistic insight into HGPS has also prompted researchers to search for some of the HGPS markers, such as the accumulation of progerin in aging adults. This is the case of Raghunath et al., who reported the reduced expression of ZMPSTE24 in smooth muscle cells of old humans compared with a younger cohort [200]. Scaffidi and Misteli also reported similar findings in fibroblasts from old adults with increased DNA damage and changes in histone modifications [201]. They also demonstrated the presence of progerin in adults, yet there was no evidence of the continuous accumulation of progerin. McClinton and Olive have also identified the accumulation of progerin in the skin and coronary arteries in older adults [202,203]. Investigating accelerated aging syndromes is pivotal in illuminating the complex interplay between the structure and function of chromatin in maintaining organismal homeostasis (Figure 4).

## 4. Future Perspective of Epigenetic Therapies and AI Technology

### 4.1. Future Perspective on Aging

Developing targeted epigenetic therapies for aging represents an exciting perspective that comes with challenges. Epigenetic drugs must have high specificity due to the potential for off-target and long-term effects. Effective therapies must selectively influence age-associated epigenetic markers while avoiding disrupting essential epigenetic functions crucial for normal cell development and specialization. Artificial intelligence (AI) is an emerging technology that holds significant promise in addressing these challenges. Advanced machine learning algorithms, capable of processing and interpreting massive volumes of biological data, are being used in drug discovery and to build predictive models of epigenetic modifications [204,205]. Epigenetic signatures, such as super-enhancers and unique histone modification patterns, can be used to determine the combinatorial functions of cell identity genes (CIGs). A Computational Epigenetic Framework for Cell Identity Gene Discovery (CEFCIG) platform has been developed, which utilizes histone codes for predicting CIGs and their master regulators with high accuracy. For instance, research has revealed that distinct regulatory mechanisms govern the expression of cell identity genes (CIGs) compared to other expressed genes, such as housekeeping genes. Specifically, super-enhancers and a unique broad pattern of H3K4me3 modification have been identified as regulators of CIGs, whereas typical enhancers and sharp H3K4me3 modifications are associated with the regulation of other expressed genes [206].

In prior work, DANPOS and DANPOS211 were introduced as pioneering tools for the analysis of genome-wide chromatin marks, encompassing aspects such as nucleosome positioning, histone modification, chromatin protein binding and chromatin openness. Building on these advancements, GridGO, a next-generation toolkit tailored for ChIP-Seq analysis, was created. This algorithm represents an innovative approach to overcome the challenges inherent in studying cell identity genes (CIGs) by automatically optimizing parameters using a grid-based parameter optimization method. This feature enables GridGO to adapt the ChIP-Seq bioinformatics pipeline to specific research objectives, thus accommodating the diverse landscape of CIG-associated chromatin marks. Moreover, six key features of the enrichment peaks were integrated: height, width, total signal, coverage, skewness and kurtosis, related to a particular type of chromatin mark at each gene. Through the integration of these tools, fundamental aspects of cell identity research are being addressed, including the elucidation of the epigenetic code governing the transcriptional regulation of CIGs and the identification of unique mechanisms by which master transcription factors regulate the network of cell identity [206].

Targeting histone acetylation and deacetylation also confers an interesting strategy, as they can regulate chromatin structure and gene expression by balancing acetylation and deacetylation, which are disrupted during aging. Compounds such as HDAC inhibitors (e.g., trichostatin A, vorinostat and valproic acid) have demonstrated anti-aging effects in various models by promoting more ‘open’ chromatin conducive to gene expression [207]. Additionally, inhibitors targeting histone methyltransferases (HMTs) and histone demethylases (HDMs) have been explored to correct aberrant histone methylation patterns associated with aging [208]. Bromodomain and extra terminal domain (BET) proteins are epigenetic ‘readers’ that recognize acetylated lysine residues on histones and recruit transcriptional machinery to specific genes. BET inhibitors, such as JQ1, inhibit these proteins and show potential in modulating inflammatory responses and cell proliferation, which are processes linked to aging and age-related diseases [209].

As mentioned above, DNA methylation patterns change significantly over time, resulting in a mix of hypermethylated and hypomethylated regions in the genome. Emerging therapeutics in this domain include DNMT inhibitors, drugs affecting histone acetylation, such as HDAC inhibitors, and molecules that specifically modulate epigenetic readers and erasers. DNMT inhibitors, such as azacytidine and decitabine, were approved for the treatment of myelodysplastic syndrome and are now being investigated for their potential to rejuvenate tissues and revert aging through methylation [210,211].

Sirtuins are a family of NAD+-dependent deacetylases implicated in lifespan extension across various species. Resveratrol, a naturally occurring sirtuin activator, and other more potent synthetic activators (e.g., SRT1720) have been investigated for their effects on lifespan and longevity [212]. Sirtuins, particularly SIRT1, are key targets due to their role in DNA repair, metabolism and stress resistance, all of which are crucial components of aging. The expression of non-coding RNAs, including microRNAs (miRNAs) and long noncoding RNAs (lncRNAs), becomes dysregulated during aging and contributes to the decline in cellular function. For example, miRNA mimics or inhibitors (antagomirs) are being developed to restore the profiles of small regulatory RNAs to those observed in younger cells. Targeting miRNAs, such as miR34a, which can influence cell senescence, DNA damage response and inflammation, is also a promising strategy [213].

The use of technology capable of mining and analyzing massive databases aiming at the identification of novel therapeutic targets for rare diseases has been observed, as seen in a recent study on HGPS by Wang et al. Gene expression profiles of GSE113648 and GSE41751 were obtained from the Gene Expression Omnibus database and subjected to analysis to identify differentially expressed genes (DEGs) between individuals with HGPS and normal controls. Gene Ontology (GO) and Kyoto Encyclopedia of Genes and Genomes (KEGG) pathway enrichment analyses were conducted to elucidate the biological processes and pathways associated with these DEGs. The analysis of DEGs revealed enrichment in various biological processes, including both positive and negative regulation of transcription from the RNA polymerase II (Pol II) promoter, cell adhesion and positive regulation of GTPase activity. Notably, Pol II transcription is remarkably active in lamin B-deficient nuclear blebs, which is characteristic of atypical progeria cells. Additionally, protein-protein interaction (PPI) networks were constructed using STRING and Cytoscape to facilitate module analysis of the identified DEGs. The Connectivity Map (cMAP) tool was employed to predict potential drugs that could reverse the expression profiles of DEGs, with the aim of identifying compounds with therapeutic potential for HGPS [214]. Module analysis of the PPI network highlighted associations between HGPS and processes involving proteoglycans in cancer and glycosaminoglycan biosynthesis, metabolism and catabolism. Previous studies have linked abnormalities in proteoglycan biosynthesis to progeroid-like symptoms in patients. In addition, O-glycosylation, the primary form of protein glycosylation, has been implicated in progeria. cMAP analysis revealed two compounds, dexibuprofen and parthenolide, which are particularly noteworthy candidates for further investigation [214].

The need for an accurate marker to identify and predict aging and diseases linked with aging is critical, as traditional chronological age falls short when accounting for the varying vulnerabilities of tissues and organs that are part of the aging process. Biological age (BA), which reflects structural and functional changes influenced by both genetic and environmental factors, is a more precise estimator that can identify individuals at risk for age-related diseases, offering an opportunity for early intervention. Some of the emerging biomarkers to calculate BA are the leukocyte telomere length [215], the use of DNA methylation clocks [216], brain imaging [217], retinal image [218] and facial analysis [219]. Most recently, the work of Wang and collaborators applied a multimodal image fusion AI model, incorporating retinal fundus, facial and tongue images, to predict BA that represents the physiological or pathological states across multiple organ systems [220]. The model optimized using a joint loss function with image detail enhancement exhibited that multimodal BA output was the most accurate in healthy populations and that BA was substantially increased in diseases and unhealthy lifestyles, proving to be a strong predictor of chronic diseases. Specifically, significant gaps between predicted retinal age and chronological age offered insights into brain health, while facial age emerged as a valuable indicator for skin health. The results concluded that the multimodal image fusion AI model outperformed predictions using the individual modalities. Furthermore, the BA model estimates closely matched the true age of healthy subjects, and it effectively highlighted the influence of chronic diseases and lifestyle factors on BA.

Personalization is another breakthrough area in which AI is striding. By analyzing individual genomic and epigenetic profiles, AI can recommend personalized therapeutic strategies tailored to each individual’s unique epigenetic landscape. This tailored approach can also reduce the risk of adverse effects and improve therapeutic outcomes. By using machine learning to monitor long-term safety, which can process extensive amounts of post-market data from electronic health records to sensor outputs, it can vigilantly detect long-term effects and ensure the ongoing safety of epigenetic therapies. These models enhance the precision of targeting specific epigenetic changes linked to the aging process, thereby guiding the development of drugs that modulate these targets with minimal off-target effects.

In the realm of drug discovery and design, AI is already screening vast libraries of compounds and predicting their interactions with predetermined targets. By simulating these interactions in silico, AI can save considerable time and resources for identifying effective drug candidates before any physical laboratory work begins. Structural bioinformatics, powered by AI, will enable researchers to understand the intricate three-dimensional structures of epigenetic proteins and design molecules that precisely fit altered versions of these proteins associated with aging.

### 4.2. Future Perspective on Cancer

Personalized therapies targeting tumor-specific factors can help improve cancer treatment success rates. Artificial intelligence, particularly machine learning (ML), is an invaluable tool in this fight. It efficiently handles big data and improves decision-making, which could revolutionize cancer care by offering precise diagnoses, prognoses and treatment modalities [221].

AI tools are particularly adept at molecular design and optimization. They can efficiently analyze the structures and properties of compounds and predict their interactions with biological targets. Various platforms and databases, such as AlphaFold2 by DeepMind, DeepChem, PubChem and ChEMBL, provide extensive resources for researchers to model compounds, target proteins and drug responses and interactions. In the realm of drug discovery, artificial neural networks (ANNs) and deep learning (DL) algorithms play a critical role in various stages [222,223,224], including the synthesis of peptides, virtual screening, toxicity prediction and the modeling of pharmacophores [225,226]. To accelerate drug design based on a target molecule structure, computational codes have been engineered to predict the three-dimensional structures of proteins [227]. Advanced algorithms like convolutional neural networks (CNN) help predict the necessary contact points between residue pairs [228,229]. Furthermore, databases cataloging protein-protein interactions, such as STRING and KEGG, are indispensable for comprehending biological systems and pinpointing new targets for drug therapy. DL methodologies enhance the prediction of protein-protein interaction interfaces, offering a substantial improvement over other ML techniques. Identifying potential druggable sites on these interfaces is critical because they contribute significantly to the binding energy [230]. Other techniques, including fragment docking and direct coupling analysis, contribute to determining actionable sites for drug design, allowing for the computational creation of small molecules that modulate these protein interaction points [231].

Moreover, the integration of bioinformatics with high-throughput epigenetic profiling has greatly advanced our understanding of gene regulatory mechanisms. Next-generation sequencing (NGS) enables precise parallel sequencing, facilitating comprehensive epigenomic investigations. ML techniques, including active learning, DL and imbalanced class learning, have been instrumental in analyzing epigenetic datasets, particularly in cancer-related studies. ML models, such as artificial neural networks and linear discriminant analysis, have demonstrated efficacy in classifying cancer cell lines based on their DNA methylation patterns, aiding in the characterization of epigenetic landscapes [232]. The identification of silencers in specific cells plays a crucial role in understanding gene expression regulation and the development of diseases such as cancer. Conventional computational methods that rely solely on DNA sequence information lack the capacity to accurately discern cell-specific silencers [233,234,235]. To address this limitation, DeepICSH, an advanced deep learning framework that integrates multiple biological data sources, utilizes a deep convolutional neural network to automatically capture biologically relevant signal combinations associated with silencers across diverse biological signals [236]. Attention mechanisms aid in scoring and visualizing these signal combinations, whereas skip connections facilitate the fusion of multilevel sequence features and signal combinations, enabling precise silencer identification within specific cells. DeepICSH demonstrates significant potential for advancing the study and analysis of silencers in complex diseases, surpassing existing methods such as gkm-SVM, DeepSilencer and SEPredict in terms of accuracy. By leveraging multi-omics data, strong and weak silencers can be identified with a high level of accuracy on independent test sets. Furthermore, specific combinations of epigenetic marks, such as H3K9me3, H3K36me3 and H3K27me3 in various cell lines have been identified as highly correlated signals for silencer identification [236].

Another helpful tool is iHMnBS, which utilizes a specialized dataset that enables the annotation of histone modifications capable of binding to various regions within DNA sequences [237]. By leveraging deep neural networks, valuable information is extracted from this extensive dataset. Through comprehensive evaluations, this feature demonstrates advanced performance compared to traditional methods, offering a reliable reference for biological experiments based on the probability of binding to modified histones at specific nucleotide positions within DNA sequences. Unlike existing methods like DeepHistone and DeepPTM [121,238], iHMnBS can detect modifications in histones binding to any segment of DNA and can predict nucleotide binding to modified histones [237].

The integration of AI-driven approaches with vast amounts of clinical and omics data has provided clinicians with novel tools for addressing these critical aspects of cancer. In breast cancer, the focus has been on using machine learning in early diagnosis and treatment, especially in integrating image recognition analysis. Many of the clinical studies include convolutional neural networks (CNN), deep convolutional neural networks (DCNN), fully convolutional networks (FCN), recurrent neural networks (RNN) and generative adversarial networks (GAN), among other modalities [239,240,241,242].

The use of images and ML algorithms were also applied to the prevention and detection of colorectal cancer using histopathological screening biopsy, which demonstrated promising outcomes. A composite algorithm was developed comprising both DL and traditional machine learning components. The DL component was founded on a faster region-based convolutional neural network (Faster-RCNN) architecture, leveraging a ResNet-101 feature-extraction backbone for glandular segmentation. The validation of this artificial intelligence (AI) model yielded a remarkable area under the curve (AUC) of 0.917, coupled with a notably high sensitivity of 97.4% for detecting high-risk indicators of cancer and dysplasia [243]. Also, a significant multicenter study utilizing randomized data underscored the pivotal role of AI assistance in colonoscopy procedures for individuals undergoing colorectal cancer screening without presenting symptoms. By building upon the success of AI-assisted image analysis in other cancer screening modalities, such as mammography and lung cancer screening, there is a growing impetus to expand the application of AI technology to endoscopic procedures within the gastrointestinal (GI) tract [244].

AI and ML technologies have also significantly affected the clinical management of colorectal cancer. Concurrently, aberrant DNA methylation patterns, particularly CpG island hypermethylation in the gene promoter regions, have emerged as key contributors to pathogenesis. The synergistic application of AI technologies and understanding DNA methylation dynamics offers new avenues for improving colorectal cancer management strategies, ultimately leading to enhanced patient outcomes and survival rates [245].

## 5. Conclusions

In summary, the dance of chromatin remodeling is pivotal to cellular integrity, guiding stem cell fate, aging and cancer development with histone modifications acting as critical conductors. The intricate control of gene expression through these epigenetic marks becomes skewed in diseases, leading to either unrestrained cell growth or an accelerated decline akin to aging. In navigating this complex genetic terrain, AI emerges as a transformative force, adept at dissecting vast layers of data to unlock the secrets of the histone code. This synergy of cutting-edge AI with epigenetic research holds the promise of groundbreaking approaches to rejuvenation and precision oncology as we look to a future where the restoration of youthful cellular function and targeted cancer therapies are within our grasp.

## Figures and Tables

**Table 1:** Histones modification in cells.

Histone	Function	Citations
H2A.Z	Active transcription, also found in premature aging.	[4,5,6]
H2A.X	Early response to double-strand breaks.	[7,8,9,10]
H3K4me3	Involved in transcription initiation, elongation and RNA splicing. Typically located at the surrounding euchromatic regions.	[11,12]
H3K9me2	Evolutionarily conserved mark of peripheral heterochromatin. Marker of heterochromatin at the nuclear periphery. Involved in the reassembly of the nuclear lamina after cellular mitosis.	[13,14]
H3K9me3	Hallmark of constitutive heterochromatin, gene silencing, epigenetic inheritance, heterochromatin assembly.	[15,16,17,18]
H3K27me3	Hallmark of facultative heterochromatin, transcription repression. Maintenance of transcriptional silencing throughout cell divisions.	[19,20]
H3K36me3	Regulate life span and active transcription.	[21,22,23]
H3K56ac	Genomic stability, chromosome segregation and cell division.	[24,25,26]
H4K20me1 H4K20me2	Associated with transcriptional activation.	[27,28]
H4K20me3	Transcription repression.	[27,29]
H4K5ac	Rapid transcription and bookmarking, memory formation	[30,31]
H4K8ac	Marker of transcriptionally active regions. Enriched in neural cells of the brain exposed to early life exercise.	[27,32,33]
H4K12ac	Associated with learning and memory, active transcription on estrogen-induced genes.	[27,34,35]
H4K16ac	Associated with transcription, DNA repair, active chromatin landscape	[36,37,38]

**Table 2:** Histone modification in stem cells.

Histone	Function	Citations
H3K4me3 H3K27ac	Maintenance of stem cell pluripotency, activated during embryonic stem cell development, global loss during differentiation of ESC to progenitor cells	[83,84,89,90,91,92,93]
H3K9me2	Maintenance of stem cell identity	[89,94]
K3K9me3	Heterochromatin formation, inhibited during embryonic stem cell development	[91,92]
H3K27me3	Loss during stem cell line commitment, inhibited during embryonic stem cell development, repression and silencing of genes associated with differentiation in stem cells.	[85,86,90,91,92,95]
H3K36me3	Embryonic stem cell differentiation towards endoderm.	[96]
H3K56ac	Human core transcriptional network of pluripotency	[97]
H4K20me3	Maintenance of stem cell self-renew	[98]

**Table 3:** Histone modification in cancer.

Histone	Function	Citations
H3K4me3	Linked to tumorigenesis, maintenance of leukemia stem cell; associated with poor prognosis of hepatocellular carcinomas	[113,114,115,116,117,118,119,120]
H3K9me2	Repressive marks; found to safeguard cancer cells from interferon pathway; found highly methylated in hepatocellular carcinomas; prevent carcinogens in normal cells.	[114,121,122,123,124]
H3K9me3	High methylation is associated with poor prognosis of hepatocellular carcinomas; prevent carcinogens in normal cells.	[123,124]
H3K27me3	Mixed function: Reduced in breast, colorectal and nasopharyngeal cancers; elevated in B-cell lymphoma (EZH2^Y641F/N^).	[108,125,126,127]
H3K27M	Hotspots mutations to the unstructured N-terminal tail of histone H3 in pediatric high-grade gliomas; lead to global reduction of H3K27me3.	[128,129,130,131]
H3K36M	Mutated in glioblastoma, induces formation of sarcomas, found prevalent in chondroblastomas, leads to global reduction of H3K36me2/3.	[131,132,133,134]
H3K79me	Active in Mixed Lineage Leukemia (MML-ENL) and hypomethylated in leukemia stem cells.	[113,135,136]
H4K16ac	Loss is a common hallmark of cancer; reduced levels in breast cancer.	[137,138]
H4K20me2	Reduction levels in renal cell cancer.	[138,139,140]
H4K20me3	Reduction levels in renal cell cancer; oncogene-induced senescence-associated proliferation arrest and tumor suppression function; poor prognosis of hepatocellular carcinoma; reduced levels in breast cancer.	[138,139,140,141,142,143]

**Table 4:** Histone modification in aging.

Histone	Function	Citations
H3K4me3	Facilitates gene expression in aging cells, globally decrease across all actively expressed genes with age.	[119,165,166,167]
H3K9me2	Hallmark of inactive euchromatin, decrease in aging	[168,169]
H3K9me3	Hallmark of constitutive heterochromatin, decrease in aging	[168,169,170]
H3K27me3	Cell-specific heterochromatin regions, regulation of lifespan.	[107,171,172]
H3K36me3	Promotes longevity, mutation reduces life span, globally decrease across all actively expressed genes with age, mark of transcribed regions.	[22,167,173,174]
H3K56ac	Mutation reduces life span	[160,170,175]
H4K16ac	Mutation reduces life span, hypoacetylation in human retinal pigment epithelium, hypoacetylated in aged epidermal basal cells.	[175,176,177]
H4K20me3	Repression is associated with cellular senescence and is involved in the control of cell senescence.	[143,155,159,160]

**Table 5:** Histone modification in progeroid syndromes.

Histone	Function	Citations
H3K9me3	Loss in Hutchinson-Gilford progeria syndrome and Werner syndrome.	[157,186,187,188,189,190]
H3K27me3	Loss in Hutchinson-Gilford progeria syndrome and Werner syndrome.	[188,190,191,192,193]
H3K36me3	Underrepresented in Hutchinson-Gilford progeria syndrome.	[192]
H4K16ac	Hypoacetylated in Hutchinson-Gilford progeria syndrome	[194]
H4K20me3	Increase in trimethylation in Hutchinson-Gilford progeria syndrome.	[177,186,187,195,196]
